# Black Currant (*Ribes nigrum* L.) and Bilberry (*Vaccinium myrtillus* L.) Fruit Juices Inhibit Adhesion of* Asaia* spp.

**DOI:** 10.1155/2016/3671306

**Published:** 2016-09-25

**Authors:** Hubert Antolak, Agata Czyzowska, Dorota Kregiel

**Affiliations:** Institute of Fermentation Technology and Microbiology, Lodz University of Technology, Wolczanska 171/173, 90-924 Lodz, Poland

## Abstract

The aim of the study was to evaluate the activity of high-polyphenolic black currant (*Ribes nigrum* L.) and bilberry (*Vaccinium myrtillus* L.) juices against bacterial strains* Asaia lannensis* and* Asaia bogorensis* isolated as spoilage of commercial soft drinks. The composition of fruit juices was evaluated using chromatographic techniques HPLC and LC-MS. The adhesion to glass, polystyrene, and polyethylene terephthalate in two different culture media was evaluated by luminometry and the plate count method. The major anthocyanins in the* V. myrtillus* were petunidin-3-glucoside, malvidin-3-glucoside, cyanidin-3-glucoside, and delphinidin-3-glucoside, while in* R. nigrum* delphinidin-3-rutinoside and cyanidin-3-rutinoside were detected. The LC-MS analysis showed presence of anthocyanins (delphinidin, cyanidin, petunidin, and malvidin derivatives), phenolic acids (chlorogenic and neochlorogenic acids), flavonols (quercetin-3-glucoside, quercetin-3-rutinoside), and flavanols (procyanidin B2 and procyanidin type A2). Additionally, in the bilberry juice A type procyanidin trimer was detected. The adhesion of* Asaia* spp. cells depended on the type of medium, carbon sources, and the type of abiotic surfaces. We noted that the adhesion was significantly stronger in minimal medium containing sucrose. The addition of bilberry and black currant juices notably reduced bacterial growth as well as cell adhesion to polyethylene terephthalate surfaces.

## 1. Introduction

Nowadays, consumers are increasingly interested in their health and expect the foods, besides possessing the sensory attractiveness, to have health-promoting effects. Numerous studies indicate that a diet rich in berries and their preserves positively affects human health. Regular consumption of fruits may delay ageing processes and reduce the risk of various illnesses, such as cancer, cardiovascular and lung diseases, rheumatoid arthritis, Alzheimer's dementia, or Parkinsonism [1–3]. Fruit berries were identified as sources of phenolic compounds like gallic and ellagic acid with potential cancer chemopreventive activity. The different bioactive phenolic compounds, including flavonoids (flavonols and flavanols), tannins (proanthocyanidins, ellagitannins, and gallotannins), stilbenoids, and phenolic acids, have received considerable interest in bearing possible relations to human health [1]. Besides health-promoting properties, polyphenols may also act as antimicrobials and antiadhesive agents in wide range of pathogens [4]. It was documented that berry extracts or juices showed strong activity against Gram negative bacteria [5, 6]. In the past decade, cranberry extracts were attracting ever-growing attention of microbiologists. It was noted that cranberry polyphenol fraction inhibits growth and adhesion of urinary tract pathogens (*Escherichia coli*,* Proteus vulgaris*),* Helicobacter pylori*, and bacterial etiological factors of oral diseases (*Streptococcus* spp.,* Propionibacterium *spp., and* Fusobacterium* spp.) [7–12].

Lately, numerous reports detailed various spoilage microorganisms in soft drinks, for example, acetic acid bacteria belonging to the genus* Asaia* [13–15]. The growth of these microorganisms causes significant changes in both microbiological and organoleptic qualities.* Asaia* spp. cells are able to grow in soft drinks supplemented with different preservatives (benzoate, sorbate, and dimethyl dicarbonate) [15]. What is more, these bacteria show strong adhesive abilities on food-contact technical materials. The biofilm formed by* Asaia* species on solid surfaces of a production line can be a source of secondary contamination of final products [16].

The initial, key step leading to biofilm formation is bacterial adhesion to the surface. This is the complex process, influenced by various physical and chemical properties of microbial cells, media, and abiotic surfaces. Among these factors, modification of media could be changed in order to prevent biofouling in soft drinks technology. New antimicrobial strategy is the use of berry juices to inhibit or reduce bacterial adhesion. The application of native and low-priced fruits with additional potential as health-promoting agents is especially interesting. Therefore, the aim of our study was to investigate antibacterial and antiadhesion activities of juices from bilberries and black currants against* Asaia* spp. cells.

## 2. Materials and Methods

### 2.1. Plant Material

The black currant (*R. nigrum* L.) and bilberry (*V. myrtillus* L.) fruits were freshly harvested from the local orchard and forests around Lodz (central Poland). The fruits were washed with sterile water, lightly air-dried, and frozen at −20°C for one month. The fresh juice was squeezed out from defrosted fruits using extractor MES3000 (Bosch, Poland). Cloudy juice was clarified using Whatman qualitative paper-filter and then by 0.45 *μ*m filtration (Filter-Bio). Immediately after preparation, the clear juice was added to the culture media to the final concentration of 10% (v/v).

### 2.2. Bacterial Strains and Culture Media

The study used the six bacterial strains:* Asaia bogorensis* ISD1 (GenBank KP234014),* A. bogorensis* ISD2 (GenBank KP234015),* A. bogorensis* FFMW (GenBank KC756841),* A. lannensis *IFMW (GenBank KP234011),* A. lannensis *IFCW (GenBank KP234012), and* A. lannensis* FMW1 (GenBank HQ917850) isolated from spoiled flavored mineral water and isotonic drinks. These strains were identified using morphological, physiological, and genetic methods described by Kregiel and coworkers [13, 17]. The obtained nucleotide sequences of 16S rRNA were deposited in GenBank (National Centre of Biotechnology Information) and the bacterial strains were deposited in the Pure Culture Collection of Industrial Microorganisms LOCK 105 at the Institute of Fermentation Technology and Microbiology, Technical University of Lodz (Poland).

The adhesion was investigated in liquid culture media: the rich GC medium (M_1_) (0.3% (w/v) peptone, 0.3% (w/v) yeast extract) and the minimal medium (M_2_) (0.3% (NH_4_)_2_PO_4_ (w/v), 0.3% KH_2_PO_4_ (w/v), 0.3% MgSO_4_  × 7H_2_O (w/v), and 0.05% (w/v) yeast extract). In both media, carbohydrates, glucose, fructose, and sucrose (2% w/v), were used as a carbon source. The sterile media (20 cm^3^) were poured aseptically into 25 cm^3^ Erlenmeyer flasks covered with a textile cloth in order to ensure aerobic conditions. Sterile carriers were placed vertically in a liquid culture medium in such a way that half of the carrier was immersed in the medium, and the other part was above the liquid.

### 2.3. Carriers

The bacterial adhesion was carried out to the polystyrene (PS) (Coveris Rigid Poland, Skierniewice) and polyethylene terephthalate (PET) (Coveris Rigid Poland, Skierniewice) slides measuring 76 × 26 mm. These materials are certified by Polish National Institute of Public Health and approved for contact with food. The white glass slides (G) (Knittel Glass, Germany) were used as the reference material. Carriers were sterilized in two-step process. First, the carriers were kept in the 70% ethanol solution for 3 hours. Subsequently, they were placed in a laminar chamber and subjected to UV irradiation for 2 hours.

### 2.4. Adhesion Analysis

Studies on the* Asaia* spp. attachment and biofilm formation were carried out in two stages. The first stage involved the selection of a culture medium, a carbon source, and an abiotic material where bacteria demonstrated the strongest adhesion abilities. In the second stage, we checked the effect of fruit juices on the growth and adhesion abilities of* Asaia* spp. For this purpose, the culture medium containing selected carbon source, with proper carrier, was supplemented with 10% (v/v) of black currant or bilberry juice.

At the beginning of the experiments culture media were inoculated with standardized bacterial suspensions, to obtain cell concentration 10^5^ ÷ 10^6^ CFU/cm^3^. The adhesion ability of the bacterial strains was evaluated according to the method described by Kregiel (2013) [16]. For luminometric tests, the carriers were removed from the culture media, washed with sterile distilled water, and swabbed with pens for ATP sampling (Merck). Measurements were made in relative light units (RLU) using a HY-LiTE® 2 luminometer (Merck). The plate count method was used in order to determine the number of cells attached to the carrier and planktonic cells in the culture medium. The carrier plate was removed from the culture medium, rinsed with sterile distilled water, and swabbed using sterile swabs for surface testing. The bacterial suspensions were vortexed with 0.1% (v/v) Tween 80 and transferred onto GC agar medium supplemented with 0.7% CaCO_3_ (w/v), and after incubation at 25°C for 92 h the colonies were counted. The number of colony forming units (CFU) per cm^3^ (of liquid media) or per cm^2^ (of carriers) was calculated. On the basis of the results, the relative adhesion coefficient *A* (%) was calculated using formula *A* (%) = (*N*_*a*_/*N*_*p*_) × 100%, where *N*_*a*_ is the number of attached cells to a carrier and *N*_*p*_ is the number of planktonic cells in the culture medium.

### 2.5. Chemical Constituent's Analysis

The organic acids and carbohydrates profiles of the tested fruit juices were determined using high performance liquid chromatography (HPLC), according to the method described by Gutarowska and Czyżowska (2009) [18]. In addition, the polyphenolic compounds were also characterized using HPLC-DAD method with a diode array detector (Finnigan Surveyor-PDA Plus detector) and a ChromQuest 5.0 chromatography software (Thermo Fisher Scientific Inc., Waltham, MA, USA) as well as using liquid chromatography mass spectrometry (LC-MS; LTQ Velos MS, Thermo Fisher Scientific) following the method described by Antolak et al. (2015) [19].

### 2.6. Statistics

Means were calculated from the data obtained from three independent experiments, and the standard deviations (SD) were calculated. The mean values of the adhesion results were compared using one-way repeated measures analysis of variance with Tukey test (ANOVA; OriginPro 8.1, OriginLab Corp., Northampton, MA). Statistical significance was set at the conventional level of 5% (*p* < 0.05).

## 3. Results and Discussion

### 3.1. Bacterial Adhesion

**Figure 1:**
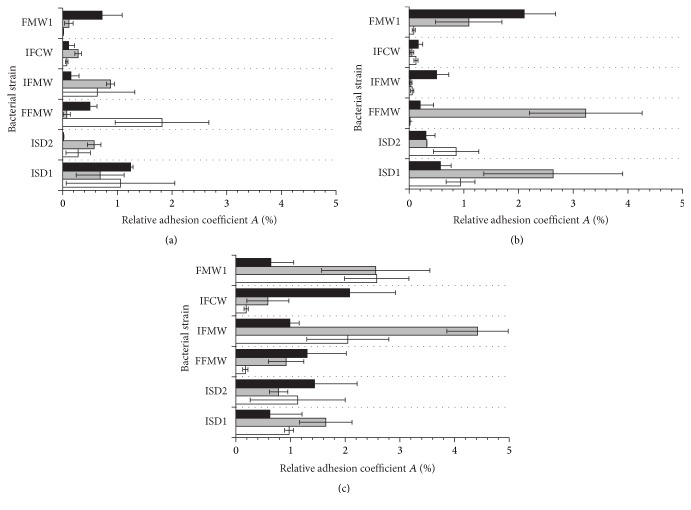
The relative adhesion coefficient *A* (%) for* A. bogorensis* (ISD1, ISD2, and FFMW) and* A. lannensis* (IFMW, IFCW, and FMW1) strains in M_1_ medium with glucose (a), fructose (b), and sucrose (c) to PET (black bars), PS (grey bars), and G (white bars).

**Figure 2:**
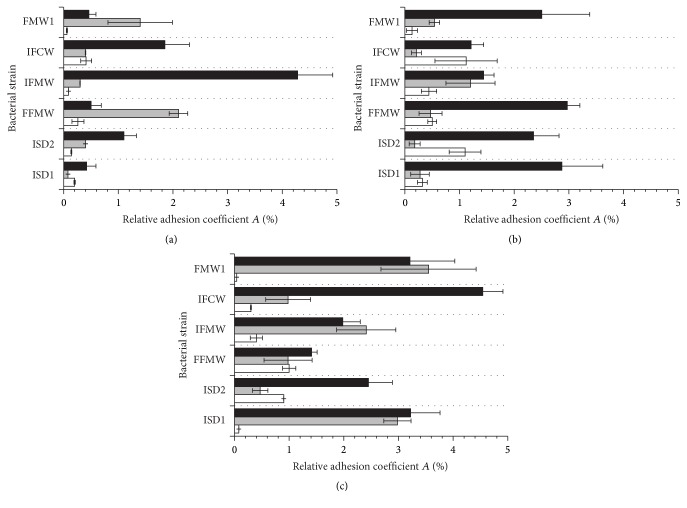
The relative adhesion coefficient *A* (%) for* A. bogorensis* (ISD1, ISD2, and FFMW) and* A. lannensis* (IFMW, IFCW, and FMW1) strains in M_2_ medium with glucose (a), fructose (b), and sucrose (c) to PET (black bars), PS (grey bars), and G (white bars).

**Figure 3:**
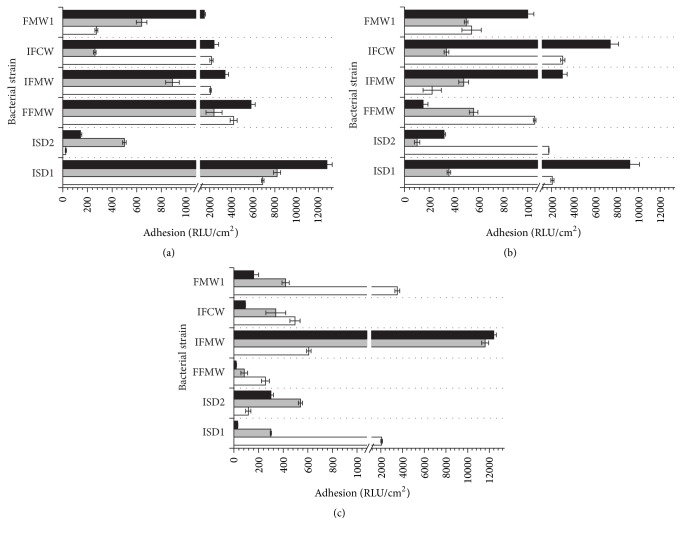
The adhesion (RLU/cm^2^) of* A. bogorensis* (ISD1, ISD2, and FFMW) and* A. lannensis* (IFMW, IFCW, and FMW1) strains in M_1_ medium with glucose (a), fructose (b), and sucrose (c) to PET (black bars), PS (grey bars), and G (white bars).

**Figure 4:**
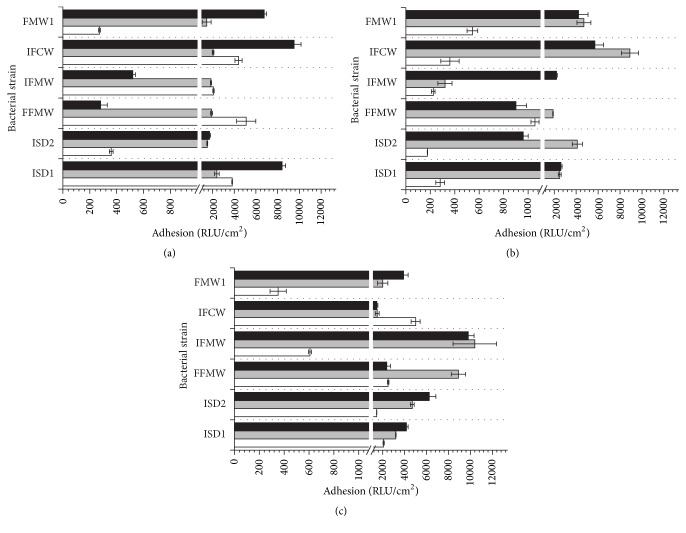
The adhesion (RLU/cm^2^) of* A. bogorensis* (ISD1, ISD2, and FFMW) and* A. lannensis* (IFMW, IFCW, and FMW1) strains in M_2_ medium with glucose (a), fructose (b), and sucrose (c) to PET (black bars), PS (grey bars), and G (white bars).

To determine the level of bacterial adhesion, two main analytical methods, namely, plate count and luminometry, were used. The evaluation of* Asaia* spp. adhesion to glass, polystyrene, and polyethylene terephthalate surfaces was carried out in rich M_1_ and minimal M_2_ medium. The influence of the carbon source for bacterial adhesion was tested in culture media supplemented with glucose, fructose, or sucrose as an only carbon source. The results of adhesion studies, expressed as relative adhesion coefficient *A* (%) for medium M_1_ and medium M_2_, are presented in Figures 1 and 2, respectively. The biofilm formation of* Asaia* strains significantly increased in culture media supplemented with sucrose (*p* < 0.05) in comparison to media containing glucose or fructose. It was noted that the minimal M_2_ medium was a more favorable environment for the* Asaia* spp. adhesion and biofilm formation compared to the rich M_1_ medium. The results for adhesion in M_2_ medium, with reference to those obtained for the adhesion in M_1_ medium, were significantly higher (*p* < 0.05). The average value of *A* (%) for cells adhesion in M_2_ medium with sucrose was 1.72 ± 0.26%, while for the same medium but with fructose and glucose the results were slight lower and equaled 1.10 ± 0.23% (*p* = 0.00001) and 0.80 ± 0.19% (*p* = 0.00004), respectively. The highest value of *A* (%) was noted for* A. lannensis* IFCW strain on PET surface, which was 4.54 ± 0.37%. Figures 3 and 4 present the luminometry results (RLU/cm^2^) obtained for bacterial adhesion in M_1_ and M_2_ media, respectively. The obtained results confirmed that the more favorable environment for biofilm formation is the minimal medium M_2_ with sucrose. Average value of the RLU for rich M_1_ medium with sucrose (1784 ± 257 RLU/cm^2^) was statistically lower (*p* = 0.001) in comparison to minimal medium M_2_ with the same carbohydrate (3923 ± 447 RLU/cm^2^).

Additionally, to assess the differences between the adhesion abilities of all bacterial strains to all tested carriers in all culture media containing different carbon sources, the mean values and standard deviations calculated from obtained results of *A* (%) (Table 1) and the RLU/cm^2^ (Table 2) were calculated. It was noted that the adhesion and biofilm formation processes were strain-dependent.* A. lannensis* strains showed slightly stronger adhesion in culture media containing sucrose. The mean *A* (%) values for* A. lannensis* strains adhesion to PET surface in culture media with sucrose were 1.23 ± 0.61% (M_1_) and 3.24 ± 1.05% (M_2_) while for the* A. bogorensis* strains 1.12 ± 0.36% (*p* = 0.05) and 2.36 ± 0.74% (*p* = 0.02) were noted, respectively.

*A. lannensis* and* A. bogorensis* were characterized by stronger adhesion properties to plastic materials in comparison to the glass surface. The average values of the relative adhesion coefficient obtained for the carriers in minimal medium M_2_ with sucrose were 0.45 ± 0.05% (G), 1.90 ± 0.23% (PS), and 2.80 ± 0.21% (PET), while for rich M_1_ medium 1.18 ± 0.71%, 1.82 ± 1.01%, and 1.17 ± 0.23% were noted, respectively. Performed ANOVA test showed that the results are statistically different. Obtained *p* values, in comparison to glass, for the M_1_ medium were 0.02 (PS) and 0.01 (PET), while the results for M_2_ medium were less than 0.01 for both PS and PET. The results of RLU measurement also showed that slightly better surface for biofilm formation in M_2_ medium with sucrose is PET.

The similar results for* Asaia* spp. adhesion were obtained by Kregiel (2013) and Kregiel et al. (2014), where, after incubation, the adhesion to plastic materials was several times higher in comparison to the glass surface [16, 17].

Of course, there are different techniques that can be used in the analysis of the microbial adhesion to abiotic surfaces, but neither method is perfect. The plate count technique in particular allows determining culturable microorganisms, while luminometric methods enable estimating total biological material on the abiotic surfaces. This approach is based on bacterial ATP quantification and can be used to evaluate not only the total number of adhering cells, but all biomass: bacteria that are able and unable to grow, extracellular polymeric substances, or adhered organic material from culture media. Thus, comparing the results of the relative coefficient *A* (%) and RLU/cm^2^, the values obtained by these two methods showed differences.

The type of material, its roughness, and hydrophobicity significantly affect bacterial attachment and biofilm development. The plastic materials used in our study were characterized by low surface energy (PET 44 mN/m at 20°C, PS 40 mN/m at 20°C) in comparison to hydrophilic glass surface (70 mN/m at 20°C) [17, 20]. What is more, studies confirmed that bacterial adhesion is influenced by many physiochemical properties of the environment, the availability and type of carbon source, and type of surface and microorganism abilities [21]. These parameters also determine the cell adhesion in industrial conditions. For example, Møretrø and Langsrud (2004) reported that food-processing environmental factors, including sugars and nutrients, had significant impacts on* Listeria monocytogenes *adhesion and biofilm formation [22]. Therefore, for the next stage of research, involving effect of berries juices on the growth and adhesion of* Asaia* spp., we choose rich M_1_ medium with glucose and minimal M_2_ medium with sucrose, respectively.

### 3.2. Chromatographic Analysis of Juices

The carbohydrate profiles of the fruit extracts indicated that the main sugars were glucose and fructose. In the bilberry juice, fructose concentration was 1.94 g/100 mL, while glucose equaled 0.76 g/100 mL. Respectively, for the black currant juice, the values were 0.60 g/100 mL and 0.54 g/100 mL. According to the literature, in the majority of native fruit juices, the content of saccharides is limited only to glucose, fructose, and sucrose. The variability of determined saccharide contents in fruit juices from berries stemmed from differences in variety, stage of ripeness, and climatic conditions [23].

**Figure 5:**
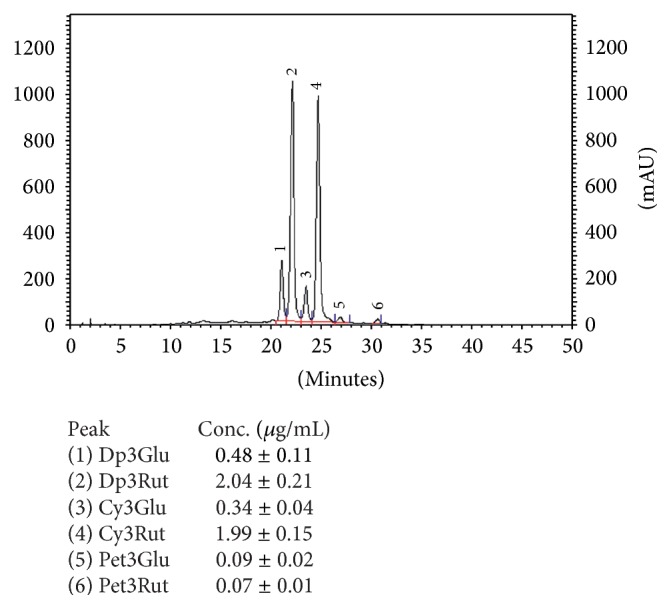
Anthocyanins profile in the* Ribes nigrum* juice.

**Figure 6:**
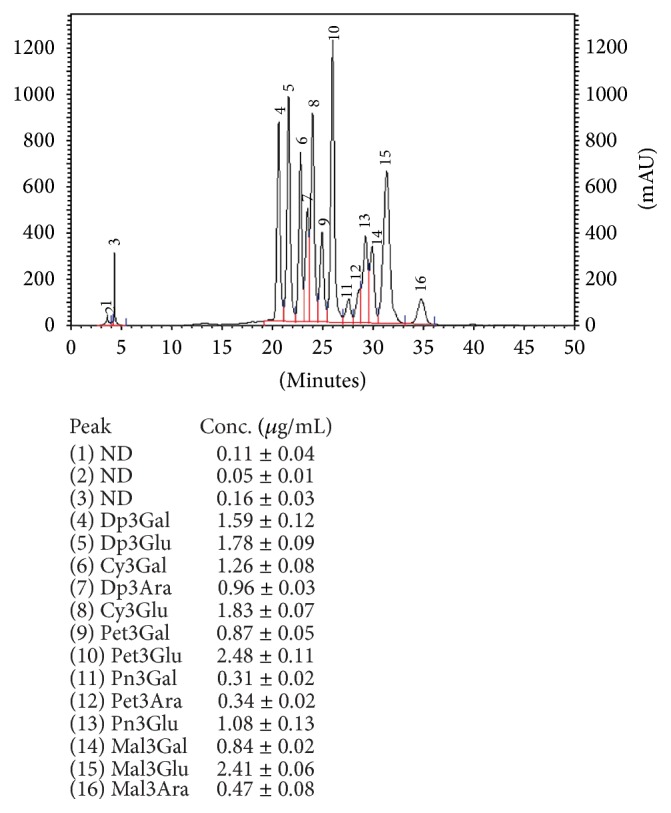
Anthocyanins profile in the* Vaccinium myrtillus* juice.

The polyphenolic profiles in fruit juices were determined using HPLC method and the results are presented in Figures 5 and 6. We noted good separation of thirteen anthocyanins in the bilberry juice while for the black currant juice we detected six defined compounds. In the bilberry juice, delphinidin (Dp), cyanidin (Cy), petunidin (Pet), peonidin (Pn), and malvidin (Mal) with galactoside (Gal), glucoside (Glu), and arabinoside (Ara) forms were detected. The results obtained for black currant juice indicate that the material is a source of delphinidin-3-glucoside, delphinidin-3-rutinoside, cyanidin-3-glucoside, and cyanidin-3-rutinoside as well as petunidin-3-glucoside and petunidin-3-rutinoside. The individual anthocyanin contents were determined according to the linear calibration curve (correlation coefficient = 0.989) and expressed as *μ*g of cyanidin-3-glucoside per one mL. The highest concentration of these compounds in the* Vaccinium myrtillus* juice was noted for petunidin-3-glucoside (2.48 *μ*g/mL) and malvidin-3-glucoside (2.41 *μ*g/mL), cyanidin-3-glucoside (1.83 *μ*g/mL), and delphinidin-3-glucoside (1.78 *μ*g/mL). The major anthocyanins in the* Ribes nigrum* juice were delphinidin-3-rutinoside (2.04 *μ*g/mL) and cyanidin-3-rutinoside (1.99 *μ*g/mL). The presence of anthocyanins was also confirmed by LC-MS (Table 3). Twenty-two compounds were detected: seven common for both juices, twelve designated only for bilberry, and three for black currant juice. Besides anthocyanins (delphinidin, cyanidin, petunidin, and malvidin derivatives), phenolic acids (chlorogenic and neochlorogenic) as well as flavonols (quercetin-3-glucoside, quercetin-3-rutinoside) and flavanols (procyanidin B2 and procyanidin type A2) were detected. Numerous studies have reported the composition of phenolic acids, anthocyanins, and flavonols in* Ribes nigrum* [24–26] and* Vaccinium myrtillus* fruits [27–30]. The bilberry fruits are a rich source of delphinidin, cyanidin, petunidin, peonidin, malvidin, and their derivatives. The anthocyanin concentration of bilberry juices ranged from 1610 to 5963 mg/L, with the mean of 3087 mg/L [30] while in the case of* R. nigrum* the average content of anthocyanin amounts to 3500 mg/L [31]. In relation to these data, black currant juice used in our study was characterized by much lower content of anthocyanins than bilberry juice, both qualitatively and quantitatively. The variations in anthocyanin profiles may be determined by genotype features of the plants and climatic conditions [28]. Despite the significant differences in the content of polyphenol compounds, juices of black currants and blueberries are rich sources of bioactive compounds that can be used as a remedy in many illnesses. It is well known that these compounds have beneficial effects in preventing cardiovascular and neurological diseases [32, 33] and possess anticancer [34, 35], anti-inflammatory [36, 37], neuroprotective [38], and antidiabetic [39] activities. The antibacterial activities of various fruit extracts on common potential pathogens including antibiotic-resistant strains were also documented [40]. Research suggests that cranberry (*Vaccinium macrocarpon*) juice, in particular, helps in maintaining the health of the urinary tract [41]. The profile of cranberry juice, being rich in A type proanthocyanidins (PACs) in contrast to the B-type PACs, presents in most other fruits [42]. PACs are colorless oligomers and polymers of flavan-3-ols that show especial antiaggregation abilities [43]. The antibacterial activity of cranberry A type proanthocyanidin was demonstrated in vitro on uropathogenic P-fimbriated* Escherichia coli* [44] and other pathogenic bacteria [7, 9]. What is interesting is that our results of LC-MS showed that bilberry juice is a source of proanthocyanidins type A and procyanidin type 2. Despite the limited literature concerning the data demonstrating the presence of type A proanthocyanidins in cranberry, some research suggests that they may also be present in wild berries. Schmidt et al. (2004) suggest that high molecular weight oligomeric proanthocyanidins from wild* Vaccinium angustifolium* exhibit strong antiproliferation activity against human prostate and mouse liver cancer cell lines [45]. Characterization of proanthocyanidins in wild blackberries was also carried out in the work of Cuevas-Rodríguez et al. (2010) [46]. Generally, the highest contents of all types of proanthocyanidins were determined in blackthorns, chokeberries, saskatoon berries, blueberries, cranberries, and lingonberries [46–52]. Moreover, it was shown that the proanthocyanidins can also be present in the bilberry fruits, chemical composition of which may be similar to that of cranberry fruit [53].

### 3.3. Growth

Due to the higher *A* (%) results and quite high RLU values, for the next stage of this study, based on the effect of fruit juices on the growth and adhesion of* Asaia* spp., we chose M_1_ medium with glucose for growth analysis and M_2_ medium with sucrose with PET carriers for adhesion investigation.

**Figure 7:**
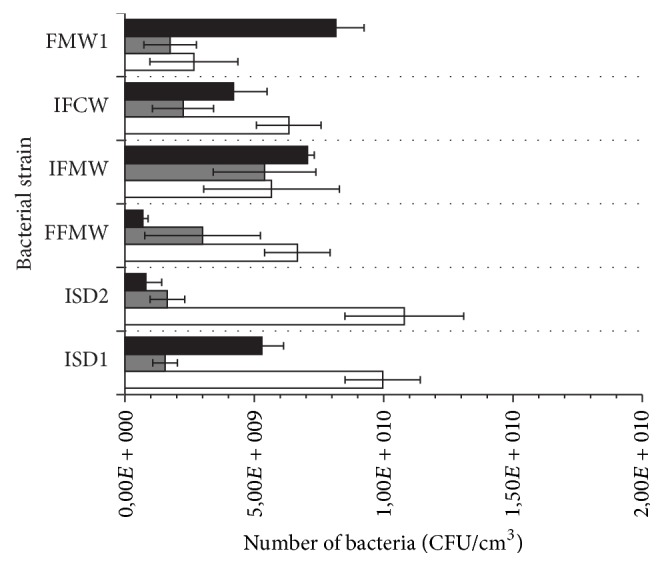
Growth of the* Asaia* spp. strains in M_1_ medium with glucose (white bars), supplemented by bilberry (grey bars) and black currant (black bars) juices.

The growth in M_1_ medium without fruit juices varied depending on the strain with mean value of 7.02 ± 2.41 × 10^9^ CFU/cm^3^ (Figure 7). After 14-day incubation, the best growth was noted for* A. bogorensis* ISD2 (1.08 ± 0.23 × 10^10^ CFU/cm^3^) and* A. bogorensis* ISD1 (9.97 ± 1.45 × 10^9^ CFU/cm^3^) while the lowest number of the bacteria was detected for* A. lannensis* FMW1 (2.67 ± 1.70 × 10^9^ CFU/cm^3^). The addition of* R. nigrum* and* V. myrtillus* juices caused a slight reduction in the number of viable bacterial cells. The average count in M_1_ medium with 10% (v/v) bilberry juice and black currant juice was 2.60 ± 1.35 × 10^9^ CFU/cm^3^ and 4.37 ± 2.85 × 10^9^ CFU/cm^3^, respectively. The obtained results suggested that* A. bogorensis* showed higher sensitivity to fruit juices than* A. lannensis* strains.

According to the literature, polyphenols from various fruit demonstrate antibacterial activities, especially against pathogenic strains:* P. aeruginosa*,* Staph. aureus*,* E. coli*,* L. monocytogenes*, and* Salmonella* spp. Polyphenols are able to suppress a number of microbial virulence factors, such as reduction of host ligands adhesion, inhibition of biofilm formation, and neutralization of bacterial toxins, and show synergism with antibiotics [54]. The activity of phenolic compounds includes interaction with microbial enzymes that are responsible for the cell growth or have direct influence on microbial metabolism by inhibition of oxidative phosphorylation [55]. In addition, the cells of Gram negative bacteria are surrounded by an outer membrane, which acts as barrier protecting against many external agents [56]. The permeability of this membrane is regulated by hydrophilic channels which generally exclude the entry of hydrophobic substances to the bacterial cell. However, some agents, including essential oils and terpenoids and other phenolic compounds, affect membrane barriers, which stimulate the penetration of bioactive agents in bacterial cells [57]. It was found that berries extracts clearly caused higher permeability of* Salmonella* spp. membranes, cell penetration, and reaction with cellular proteins [58]. According to Nohynek et al. (2006), the activity of polyphenolic compounds from berry fruits may be the result of multiple mechanisms and synergies due to the presence of various bioactive compounds [56]. In Puupponen-Pimiä et al. (2001) study, extracts from blueberry and black currant fruits were checked against pathogenic Gram negative and Gram positive bacteria [5]. It was shown that anthocyanins (pelargonidin, cyanidin) as well flavonols (myricetin) showed inhibitory effect against Gram negative cells of* E. coli* and* Salmonella* spp. Phenolic extracts containing tannins and their derivatives showed strong antibacterial effect against* Staph. aureus*,* H. pylori*,* C. perfringens*,* B. cereus*,* Klebsiella* spp., and* Proteus* spp. [56, 58]. However, the knowledge about the effect of fruit phenolics on food spoilage bacteria is still limited.

### 3.4. Biofouling

It is well known that luminometric measurements in an environment of fruit juices that are rich in polyphenols may carry a margin of error. Luminometry is based on the reaction of enzymatic oxidation of luciferin to oxyluciferin and the presence of antioxidants can influence the final results. It has been documented that polyphenols present in green tea can inhibit the enzymatic activities [59]. Therefore, in the light of that fact, we used two different methods to assess the adhesion of cells to PET surface: luminometry and plate count technique.

**Figure 8:**
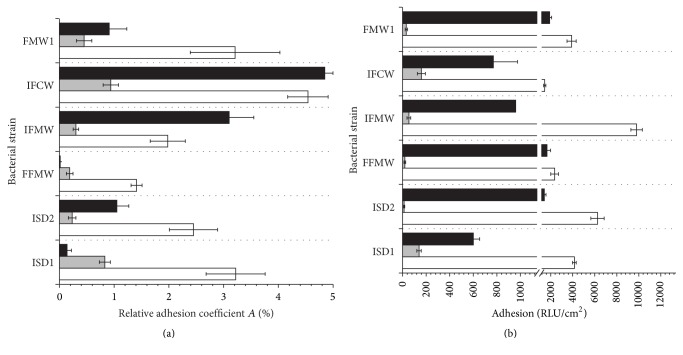
Adhesion of the* Asaia* spp. strains to PET carrier in M_2_ medium with sucrose (white bars) supplemented by bilberry (grey bars) and black currant (black bars) juices, evaluated by plate count method (a) and luminometry (b).

The effect of the bilberry and black currant juices on the adhesion properties of* Asaia* spp. was performed during cultivation in M_2_ medium with PET carriers. Results, expressed as adhesion relative coefficient *A* (%) and RLU/cm^2^, were presented in Figures 8(a) and 8(b), respectively. The coefficient *A* (%) calculated for the sixth day of incubation with 10% (v/v) juice showed significant decrease in the adhesion and biofilm formation (Figure 8(a)). This parameter for cell adhesion with bilberry juice ranged from 0.19 ± 0.11% to 0.94 ± 0.59%, while for black currant juice the values were 0.01 ± 0.009% to 4.85 ± 0.41%. The results were 4 ÷ 11 times lower in comparison to the control sample without* V. myrtillus* juice. Luminometric results (RLU/cm^2^) also confirmed significant reduction of adhesion (Figure 8(b)). There were statistically significant differences between the control samples and cultures with fruit juices (*p* < 0.05). Additionally, the differences were noted for antiadhesive activities of tested juices. The values ranged from 1460 ± 102 RLU/cm^2^ to 9800 ± 520 RLU/cm^2^ (Av = 4252 ± 2748 RLU/cm^2^) for the control sample and for adhesion in the presence of* V. myrtillus* and* R. nigrum* from 14 ± 5 RLU/cm^2^ to 160 ± 34 RLU/cm^2^ (Av = 70 ± 58 RLU/cm^2^) and from 600 ± 54 RLU/cm^2^ to 1900 ± 187 RLU/cm^2^, respectively (Av = 1218 ± 474 RLU/cm^2^). Thus, bilberry juice inhibited biofouling of all tested* Asaia* spp. bacteria, while in the presence of black currant juice we noted the antiadhesive effect for* A. bogorensis* strains in particular.

The use of fruit juice not only brings antiadhesive effects, but also has other health benefits. The prohealth action of berry juices has been known in folk medicine. However, antiadhesive properties of fruit juices were documented scientifically mainly for cranberry (*Vaccinium macrocarpon*) [17, 41, 54]. The effect of blueberry constituents on the adhesion of* Staph. mutans* was also documented [60]. The recent studies are related to the effect of cranberry juice on the growth and adhesion abilities of bacteria* Asaia* spp. It was documented that, in the presence of cranberry juice, the attachment of* A. bogorensis* cells to plastic surfaces was significantly lower [19]. However, the mechanisms by which cranberry extracts are effective as antiadhesive agent have not been fully established yet. It is believed that there are two main compounds involved in the inhibition of bacterial attachment: fructose blocking bacterial type 1 fimbriae and proanthocyanidins which bind with type P fimbriae, preventing cells adhesion [41, 61]. The chromatographic analysis of the polyphenols in* V. macrocarpon* confirmed the presence of type A proanthocyanidin [19, 62]. Thus, we can assume that type A proanthocyanidins present in berries may show an antiadhesive effect to* Asaia* spp. cells.

## 4. Conclusions

The results presented in this study suggest that bilberry and black currant juices show high antiadhesive and antibacterial activity against food-spoiled bacteria belonging to the genus* Asaia*. Particularly* V. myrtillus* juice characterized by a higher content of polyphenols including A type proanthocyanidin showed strong antiadhesive and bacteriostatic properties. The high content of bioactive compounds with proven health-promoting properties makes them a valuable supplement of soft drinks, as well as interesting alternative to artificial additives to keep the microbial stability of final products.

## Figures and Tables

**Table 1:** Adhesion of the *Asaia *spp. strains reported as relative coefficient *A* (%) in M_1_ and M_2_ media with carbohydrates as a carbon source. The mean values of the adhesion results were compared using one-way repeated measures ANOVA with Tukey test. Two results of *p* values were obtained: *p*_1_ – *p* value obtained by the comparison of the *A* (%) results within a species to the results for M_1_ with glucose and glass; *p*_2_ – *p* value obtained by the comparison of the *A* (%) between *Asaia bogorensis* and *Asaia lannensis*. Statistical significance was set at the conventional level of 5% (*p* < 0.05).

	Species											
	*A. bogorensis*						*A. lannensis*					
	Carbon source											
	Glucose		Fructose		Sucrose		Glucose		Fructose		Sucrose	
	Medium											
	M_1_	M_2_	M_1_	M_2_	M_1_	M_2_	M_1_	M_2_	M_1_	M_2_	M_1_	M_2_
Surface												

G	1.05 ± 0.63 *p*_2_ = 0.11	0.20 ± 0.05 *p*_1_ = 0.11 *p*_2_ = 0.04	0.60 ± 0.42 *p*_1_ = 0.02 *p*_2_ = 0.14	0.64 ± 0.33 *p*_1_ = 0.03 *p*_2_ = 0.14	0.76 ± 0.42 *p*_1_ = 0.01 *p*_2_ = 0.04	0.66 ± 0.41 *p*_1_ = 0.09 *p*_2_ = 0.05	0.24 ± 0.28 *p*_2_ = 0.11	0.19 ± 0.16 *p*_1_ = 0.15 *p*_2_ = 0.04	0.09 ± 0.03 *p*_1_ = 0.21 *p*_2_ = 0.14	0.56 ± 0.41 *p*_1_ = 0.14 *p*_2_ = 0.14	1.60 ± 1.03 *p*_1_ = 0.15 *p*_2_ = 0.04	0.25 ± 0.16 *p*_1_ = 0.23 *p*_2_ = 0.05

PS	0.44 ± 0.27 *p*_1_ = 0.04 *p*_2_ = 0.11	0.86 ± 0.89 *p*_1_ = 0.19 *p*_2_ = 0.25	2.06 ± 1.25 *p*_1_ = 0.14 *p*_2_ = 0.15	0.31 ± 0.12 *p*_1_ = 0.12 *p*_2_ = 0.09	1.11 ± 0.38 *p*_1_ = 0.05 *p*_2_ = 0.12	1.48 ± 1.08 *p*_1_ = 0.11 *p*_2_ = 0.09	0.42 ± 0.33 *p*_1_ = 0.26 *p*_2_ = 0.11	0.70 ± 0.50 *p*_1_ = 0.07 *p*_2_ = 0.25	0.39 ± 0.50 *p*_1_ = 0.16 *p*_2_ = 0.15	0.65 ± 0.41 *p*_1_ = 0.20 *p*_2_ = 0.09	2.52 ± 1.57 *p*_1_ = 0.16 *p*_2_ = 0.12	2.31 ± 1.05 *p*_1_ = 0.08 *p*_2_ = 0.09

PET	0.59 ± 0.51 *p*_1_ = 0.13 *p*_2_ = 0.15	0.67 ± 0.30 *p*_1_ = 0.02 *p*_2_ = 0.11	0.36 ± 0.16 *p*_1_ = 0.08 *p*_2_ = 0.14	2.73 ± 0.27 *p*_1_ = 0.02 *p*_2_ = 0.02	1.12 ± 0.36 *p*_1_ = 0.04 *p*_2_ = 0.05	2.36 ± 0.74 *p*_1_ = 0.01 *p*_2_ = 0.02	0.33 ± 0.28 *p*_1_ = 0.09 *p*_2_ = 0.15	2.20 ± 1.58 *p*_1_ = 0.20 *p*_2_ = 0.11	0.92 ± 0.84 *p*_1_ = 0.17 *p*_2_ = 0.14	1.72 ± 0.56 *p*_1_ = 0.03 *p*_2_ = 0.02	1.23 ± 0.61 *p*_1_ = 0.08 *p*_2_ = 0.05	3.24 ± 1.05 *p*_1_ = 0.03 *p*_2_ = 0.02

**Table 2:** Adhesion of the *Asaia *spp. strains, reported in RLU/cm^2^, in M_1_ and M_2_ media with carbohydrates as a carbon source. The mean values of the adhesion results were compared using one-way repeated measures ANOVA with Tukey test. Two results of *p* values were obtained: *p*_1_ – *p* value obtained by the comparison of the RLU/cm^2^ results within a species to the results for M_1_ with glucose and glass; *p*_2_ – *p* value obtained by the comparison of the RLU/cm^2^ between *Asaia bogorensis* and *Asaia lannensis*. Statistical significance was set at the conventional level of 5% (*p* < 0.05).

	Species											
	*A. bogorensis*						*A. lannensis*					
	Carbon source											
	Glucose		Fructose		Sucrose		Glucose		Fructose		Sucrose	
	Medium											
	M_1_	M_2_	M_1_	M_2_	M_1_	M_2_	M_1_	M_2_	M_1_	M_2_	M_1_	M_2_
Surface												

G	3701 ± 2821 *p*_2_ = 0.12	3086 ± 2000 *p*_1_ = 0.17 *p*_2_ = 0.01	1632 ± 428 *p*_1_ = 0.12 *p*_2_ = 0.09	504 ± 393 *p*_1_ = 0.18 *p*_2_ = 0.13	817 ± 894 *p*_1_ = 0.22 *p*_2_ = 0.13	2010 ± 432 *p*_1_ = 0.12 *p*_2_ = 0.08	1504 ± 871 *p*_2_ = 0.12	2251 ± 1690 *p*_1_ = 0.16 *p*_2_ = 0.01	1269 ± 1259 *p*_1_ = 0.16 *p*_2_ = 0.09	376 ± 131 *p*_1_ = 0.07 *p*_2_ = 0.13	1541 ± 1400 *p*_1_ = 0.01 *p*_2_ = 0.13	1987 ± 2133 *p*_1_ = 0.21 *p*_2_ = 0.08

PS	3700 ± 3375 *p*_1_ = 0.22 *p*_2_ = 0.22	1937 ± 364 *p*_1_ = 0.13 *p*_2_ = 0.01	340 ± 187 *p*_1_ = 0.19 *p*_2_ = 0.05	2830 ± 932 *p*_1_ = 0.05 *p*_2_ = 0.13	308 ± 186 *p*_1_ = 0.17 *p*_2_ = 0.36	5600 ± 2412 *p*_1_ = 0.06 *p*_2_ = 0.04	597 ± 259 *p*_1_ = 0.07 *p*_2_ = 0.22	1783 ± 249 *p*_1_ = 0.05 *p*_2_ = 0.01	440 ± 71 *p*_1_ = 0.08 *p*_2_ = 0.05	4640 ± 3503 *p*_1_ = 0.13 *p*_2_ = 0.13	4120 ± 5289 *p*_1_ = 0.30 *p*_2_ = 0.36	4633 ± 4082 *p*_1_ = 0.19 *p*_2_ = 0.04

PET	6247 ± 5177 *p*_1_ = 0.22 *p*_2_ = 0.16	3463 ± 3539 *p*_1_ = 0.23 *p*_2_ = 0.02	3223 ± 4227 *p*_1_ = 0.28 *p*_2_ = 0.16	1487 ± 788 *p*_1_ = 0.17 *p*_2_ = 0.01	817 ± 130 *p*_1_ = 0.18 *p*_2_ = 0.40	4252 ± 1591 *p*_1_ = 0.04 *p*_2_ = 0.05	2433 ± 776 *p*_1_ = 0.07 *p*_2_ = 0.16	5590 ± 3757 *p*_1_ = 0.11 *p*_2_ = 0.02	3800 ± 2673 *p*_1_ = 0.15 *p*_2_ = 0.16	4033 ± 1434 *p*_1_ = 0.04 *p*_2_ = 0.01	4217 ± 5786 *p*_1_ = 0.32 *p*_2_ = 0.40	5053 ± 3501 *p*_1_ = 0.13 *p*_2_ = 0.05

**Table 3:** Bioactive compounds in bilberry and black currant juices.

RT (min)	*λ*_max_ (nm)	[M − H]^−^	Fragment ions	Compound	*V. myrtillus*	*R. nigrum*
8.48	244, 323	353	191, 179	Neochlorogenic acid	**+**	**+**
9.06	244, 330	355	191	Chlorogenic acid	−	**+**
9.62	223, 280	463	301	Delphinidin-3-galactoside	**+**	−
9.73	246, 330	355	179, 163	Caffeoyl hexose	**+**	**+**
11.01	522	341	179	Dicaffeic acid	**+**	−
11.22	278, 521	463	301	Delphinidin-3-glucoside	**+**	**+**
11.65	224, 522	609	301, 406	Delphinidin-3-rutinoside	−	**+**
13.47	280, 520	447	285	Cyanidin-3-glucoside	**+**	**+**
15.60	280, 521	477	315	Petunidin-3-galactoside	**+**	**+**
15.63	236, 279	577	407	Procyanidin B2	**+**	−
15.93	236, 280	575	377, 395, 449	Procyanidin A2	**+**	−
16.04	272, 520	477	315	Petunidin-3-glucoside	**+**	−
20.78	260, 352	479	317	Myricetin-3-galactoside	−	**+**
20.84	254, 354	461	301	Quercetin-3-glucoside	**+**	**+**
21.20	276, 527	491	329	Malvidin-3-galactoside	**+**	**+**
21.47	233, 279	866	577, 451	B-type procyanidin trimer	**+**	−
25.34	233, 280	863	573, 411	A type procyanidin trimer	**+**	−
26.45	230, 278	1152	861, 577	A type procyanidin tetramer	**+**	−
28.59	261, 352	479	317	Myricetin-3-glucoside	**+**	−
28.68	233, 279	489	285	Cyanidin-6-acetyl-3-glucoside	**+**	−
30.34	281, 521	505	301	Delphinidin-6-acetyl-3-glucoside	**+**	−
33.71	258, 354	609	301	Quercetin-3-rutinoside	**+**	−
